# Transformed MDCK cells secrete elevated MMP1 that generates LAMA5 fragments promoting endothelial cell angiogenesis

**DOI:** 10.1038/srep28321

**Published:** 2016-06-21

**Authors:** Shashi K. Gopal, David W. Greening, Hong-Jian Zhu, Richard J. Simpson, Rommel A. Mathias

**Affiliations:** 1 Department of Biochemistry and Genetics, La Trobe Institute for Molecular Science, La Trobe University, Melbourne, Victoria 3086, Australia; 2 Department of Surgery, The University of Melbourne, Royal Melbourne Hospital, Melbourne, Victoria 3050, Australia; 3 Department of Biochemistry and Molecular Biology, Biomedicine Discovery Institute, Monash University, Clayton, Victoria 3800, Australia; 4 Department of Microbiology, Biomedicine Discovery Institute, Monash University, Clayton, Victoria 3800, Australia

## Abstract

Epithelial-mesenchymal transition (EMT) enhances the migration and invasion of cancer cells, and is regulated by various molecular mechanisms including extracellular matrix metalloproteinase (MMP) activity. Previously, we reported transformation of epithelial Madin-Darby canine kidney (MDCK) cells with oncogenic H-Ras (21D1 cells) induces EMT, and significantly elevates MMP1 expression. To explore the biological significance, in this study we characterized 21D1 cells with knocked-down MMP1 expression (21D1^−MMP1^). MMP1 silencing diminished 21D1 cell migration, invasion and anchorage-independent growth *in vitro*. Additionally, 21D1^−MMP1^ cells displayed reduced tumour volume when grown as *in vivo* subcutaneous xenografts in mice. Depletion of MMP1 lowered the ability of the cellular secretome (extracellular culture medium) to influence recipient cell behaviour. For example, supplementation with 21D1 secretome elevated cell migration of recipient fibroblasts, and enhanced endothelial cell angiogenesis (vessel length and branching). By contrast, 21D1^−MMP1^ secretome was less potent in both functional assays. We reveal laminin subunit alpha-5 (LAMA5) as a novel biological substrate of MMP1, that generates internal and C-terminal proteolytic fragments in 21D1 secretome. Furthermore, antibody-based inhibition of integrin αvβ3 on endothelial cells nullified the angiogenic capability of 21D1 secretome. Therefore, we report this as a new VEGF-independent mechanism that oncogenic cells may employ to promote tumour angiogenesis.

Epithelial mesenchymal transition (EMT) is characterized by a shift in cellular plasticity whereby epithelial cells acquire mesenchymal traits that include spindle-shaped morphology, and increased cell migration and invasion^1^,^2^. EMT is thought to promote various stages of the metastatic cascade; a process governing passage of primary tumour cells to a distant site for colonization and secondary tumour growth^3^. In the tumour microenvironment (TM), extracellular proteases exert pleiotropic effects that include EMT regulation, invasion, angiogenesis, growth factor signalling and extracellular matrix (ECM) remodelling^4^,^5^,^6^. Collectively, cancer-associated proteases enhance metastatic progression, however, not all the molecular mechanisms have been defined, including many protease-substrate interactions^7^.

Matrix metalloproteinases (MMPs) are a family of zinc-dependent endopeptidases that have been implicated in various pathological conditions including tissue remodelling, organ development and carcinogenesis^8^. An assortment of MMPs that include MMP-1,-2,-3,-7,-9,-11 and -14 exhibit elevated expression across many human tumours^9^, and their functional modes of action are starting to be revealed. For example, MMP2 and MMP9 have been shown to be involved in the degradation of basement membrane constituents during colorectal tumourigenesis^10^, generating a passage for cell motility and invasion. ECM degradation by extracellular proteases is also known to generate bioactive protein fragments, and release growth factors^11^. Laminin-5 (composed of α3β3γ2 chains) is a well-known ECM substrate processed by a variety of MMPs including MMP-2, -7, -14, and -19. Its cleavage has been shown to promote migration of keratinocytes, breast epithelial and breast carcinoma cells, and colon carcinoma and prostate cancer cells^12^,^13^,^14^,^15^,^16^. Thus, MMP specificity for the various laminin heterotrimers are beginning to emerge, however many enzyme-substrate relationships remain to be characterised.

MMP1 is an interstitial collagenase secreted by a variety of cells such as fibroblasts, endothelial and inflammatory cells, and exert paracrine and autocrine effects in the microenvironment during cancer progression^17^,^18^,^19^. Depth grading of tumour invasion and lymph node metastasis in human colorectal tumours correlate with strong expression of MMP1^6^. Notably, MMP1 was identified to be a novel downstream target of TWIST1, implicated in facilitating invasion in human melanoma cells^20^. The stable expression of the active form of MMP1 was found to promote melanoma growth through the generation of active TGF-β, an inducer of EMT^21^.

Importantly, MMP1 can directly cleave fibrillar collagens and several fundamental ECM constituents such as elastin, fibronectin, aggrecan and versican^22^,^23^,^24^. MMP1 has been identified to proteolytically activate G protein coupled receptor (PAR1) and facilitate tumour invasion^25^. Furthermore, a MMP1/PAR1 axis was found to facilitate melanoma invasion, tumour growth and metastasis^26^. Signalling precursors that include pro –TNF α can also be shed from the cell surface by MMP1^8^,^27^, and MMP1 in conjunction with ADAMTS1 was found to engage EGF-like growth factors (AREG, HB-EGF and TGF-α) and orchestrate osteolytic signalling and bone metastasis^28^.

Towards identifying novel enzyme-substrate interactions occurring within the extracellular microenvironment, we have profiled secretome, exosome, and plasma membrane protein expression in MDCK cells transformed with oncogenic H-Ras (21D1 cells)^29^,^30^,^31^,^32^,^33^. We have previously reported extensive ECM remodelling, and the salient finding was the significant expression of MMP1 in the 21D1 secretome^30^,^32^. To directly explore the functional significance, in the current study we generated 21D1 cells with knock-down MMP1 levels (21D1^−MMP1^), and examined changes to their cellular oncogenicity, and secretome protein expression perturbations. Given MMP1 is known to be involved in vascular remodelling and angiogenesis, we investigated the functional effects that loss of MMP1 expression had on recipient cells in the TM, including fibroblast and endothelial cells.

## Results

We have previously reported that 21D1 cells display mesenchymal-like cell properties, and have elevated levels of MMP1 in the extracellular secretome^30^,^32^. In this study, we depleted MMP1 from the secretome of 21D1 cells, and examined the functional consequences exerted in the extracellular microenvironment.

### MMP1 silencing diminishes 21D1 cell migration, invasion and anchorage-independent growth

**Figure 1:**
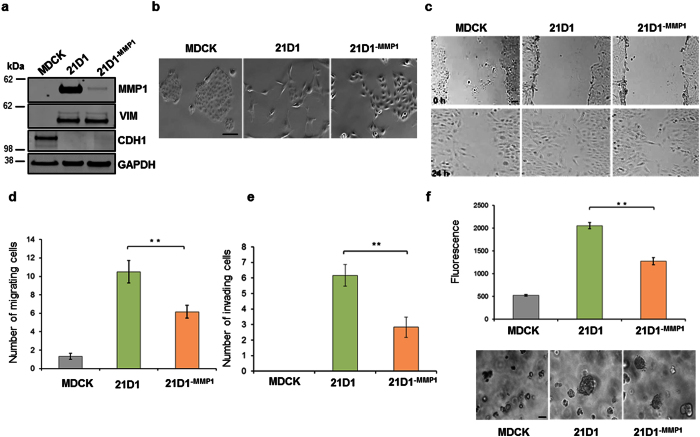
MMP1 silencing diminishes 21D1 cell migration, invasion and anchorage- independent growth. **(a)** MDCK, 21D1 and 21D1^−MMP1^ cellular lysates were immuno-blotted for MMP1, VIM, CDH1 expression, and GAPDH as the loading control. **(b)** Cell scattering was assessed by phase contrast microscopy (10x magnification) (Scale bar = 30 μm, *n* = 3). **(c)** Wound-healing migration assays were performed on sub- confluent cell monolayers and cells imaged after 24 hr by phase contrast microscopy (Scale bar = 50 μm, *n* = 3). (**d)** Transwell cell migration assays were performed using 8.0 μm membrane inserts over 24 hr. Transversing cells were stained with DAPI and counted (*n* = 3; average ± sem; **P < 0.01). **(e)** Cell invasion was assessed by coating transwells with matrigel (1 mg/ml). After 24 hr, invading cells were stained with DAPI and counted (*n* = 3; average ± sem; **P < 0.01). **(f)** Soft agar growth assays were performed using 2,500 cells which were inoculated and grown for 7 days, and then stained with CyQuant GR. (Scale bar = 100 μm, *n* = 3). Images are representative (*n* = 3; average ± sem; **P < 0.01).

Expression of miR-MMP1 in 21D1 cells (21D1^−MMP1^ cells) significantly knocked-down the expression of MMP1 (Fig. 1a). Reduced MMP1 expression did not alter expression of EMT markers E-cadherin and vimentin (VIM) (Fig. 1a). Additionally, 21D1^−MMP1^ cells retained their elongated spindle-shaped morphology, however, the degree of cellular scattering was diminished (Fig. 1b). Characterization of metabolic activity and growth rate in 21D1^−MMP1^ cells showed no significant alterations, compared to parental 21D1 cells (Supplemental Fig. 1A,B). Wound-healing assays and transwell motility assays revealed that 21D1^−MMP1^ cells have impaired cell migration, compared to 21D1 cells (Fig. 1c,d). Similarly, the invasiveness of 21D1^−MMP1^ cells was impaired (Fig. 1e), as was their ability to grow via anchorage independence (Fig. 1f, top panel). Comparison of number of colonies and colony size highlighted a significant reduction in 21D1^−MMP1^ cells, compared to 21D1 cells (Fig. 1f, bottom panel). These results demonstrate that knock-down of MMP1 expression in 21D1 cells reduces their oncogenic potential.

### MMP1 knock-down attenuates 21D1 cell tumour xenografts in mice

**Figure 2:**
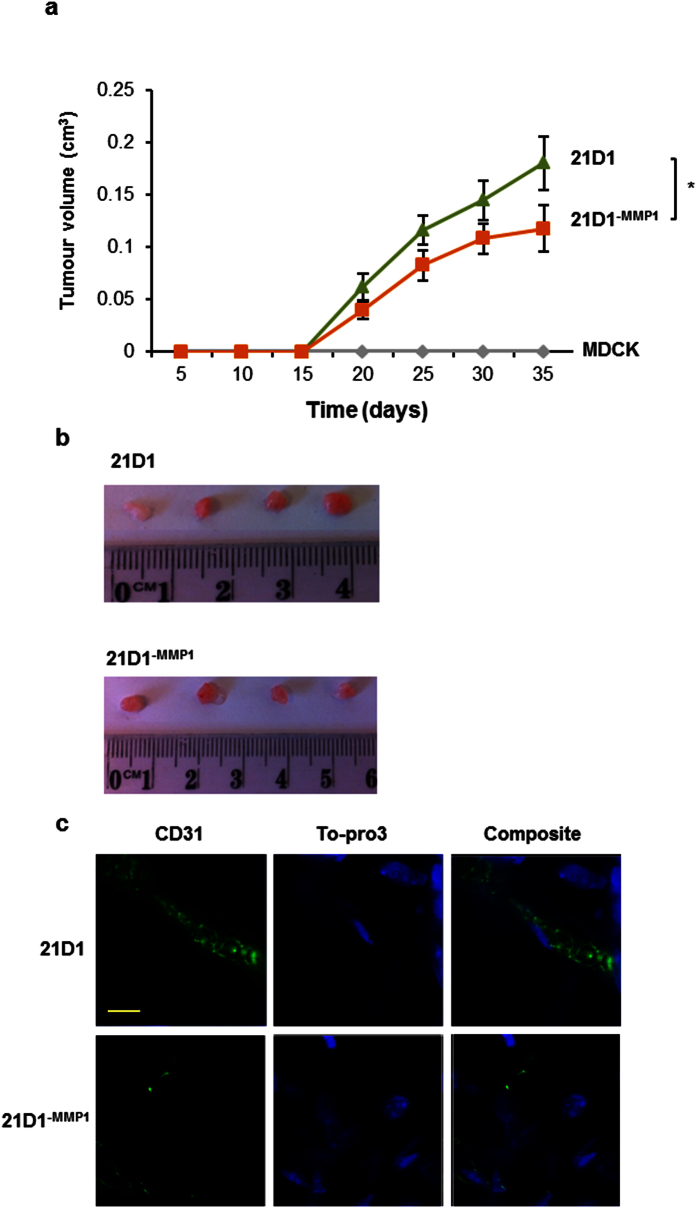
MMP1 knockdown reduces *in vivo* 21D1 tumour xenograft growth. **(a)** 1 × 10^6^ cells/site were injected subcutaneously into NOD/SCID mice on both inguinal regions. Tumour volumes were measured at indicated times (*n* = 8; average tumour volume ± sem; *P < 0.05). **(b)** Tumours were excised after 5 weeks, and representative images are shown (*n* = 8). **(c)** Excised tumours were sectioned, and immuno-histochemistry performed for CD31 expression (green) (Scale bar = 10 μm, *n* = 3).

With 21D1^−MMP1^ cells displaying reduced oncogenicity *in vitro*, we next measured the impact of MMP1 knock-down on 21D1 cell tumour xenografts *in vivo*. 1 × 10^6^ cells from each of the three lines were subcutaneously injected into NOD/SCID mice, and tumour volumes monitored over 5 weeks (Fig. 2a). MDCK cells did not establish a viable xenograft, however, tumour growth curves indicate that MMP1 silencing significantly reduced xenograft size (Fig. 2a). Representative end-point tumours are shown in Fig. 2b, and xenograft sectioning and immuno-histochemistry revealed strong staining of CD31 in 21D1 xenografts (Fig. 2c, top panel). By contrast, 21D1^−MMP1^ xenografts showed much lower expression of CD31, indicating less endothelial cell infiltration (Fig. 2c, bottom panel), and possibly lower tumour angiogenesis leading to their smaller size. Our results suggest that elevated expression of MMP1 *in vivo* promotes tumour development, and we hypothesised that this may be via regulation of various cells within the tumour microenvironment (fibroblasts and endothelial cells).

### Recipient fibroblast cell migration is hindered when supplemented with 21D1^−MMP1^ cell-derived secretome

**Figure 3:**
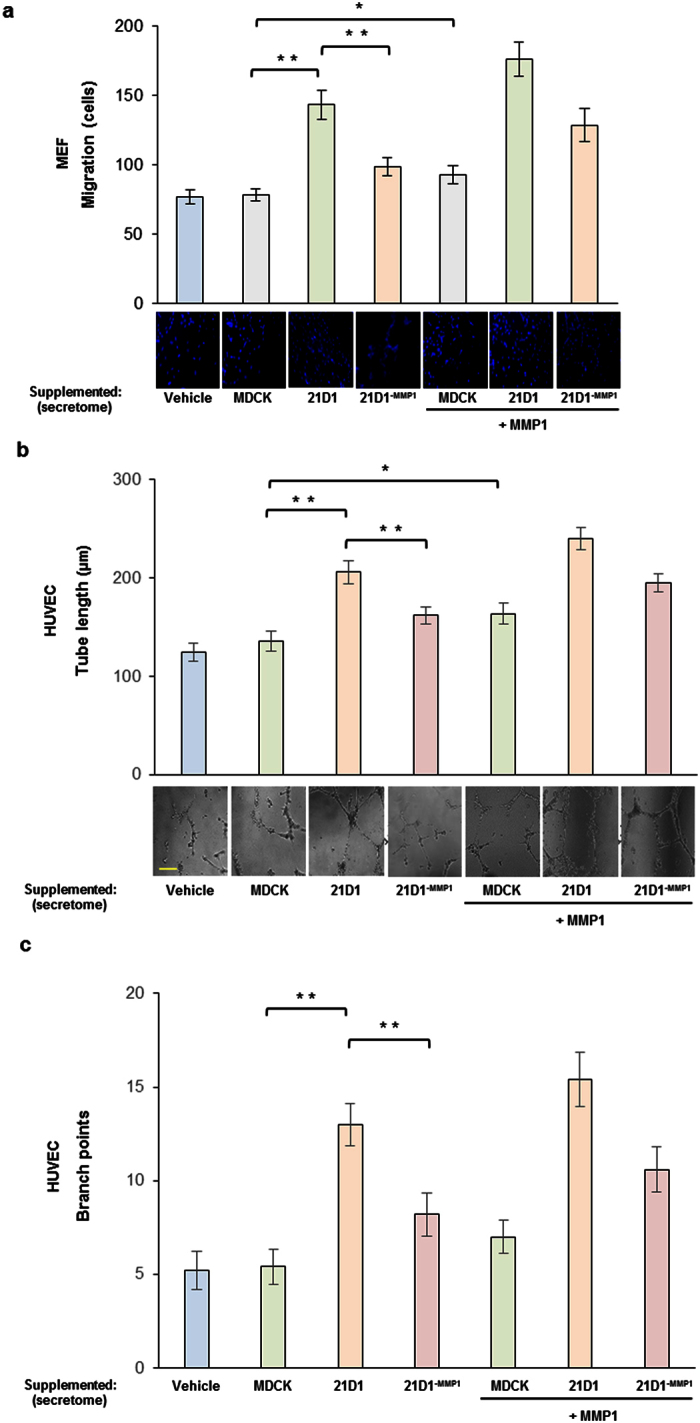
MMP1 expression in the secretome modulates recipient cell function. **(a)** MEF (5 × 10^4^) cell migration over 24 hr was assessed by transwell assay following supplementation with either MDCK, 21D1 or 21D1^−MMP1^ secretome (30 μg), plus or minus activated recombinant MMP1 (100 ng). Transversing cells were stained with DAPI, imaged and counted (*n* = 3; average ± sem; *P < 0.05, **P < 0.01). **(b**,**c)** HUVEC (7 × 10^4^) cell angiogenesis was assessed following supplementation with either MDCK, 21D1 or 21D1^−MMP1^ secretome (30 μg), plus or minus activated recombinant MMP1 (100 ng). Cells were analyzed for tube length **(b)** and branch points **(c)** (Scale bar = 50 μm) (*n* = 3; average ± sem; *P < 0.05, **P < 0.01). Vehicle control is DMEM only.

To explore the potential regulation of cells in the TM (fibroblasts) by oncogenic cells undergoing EMT, we collected secretome (Supplemental Fig. 2) from our three cell lines, and supplemented mouse embryonic fibroblasts (MEFs). Recipient MEFs cell migration was monitored using the transwell assay, which demonstrated that addition of MDCK secretome did not alter MEF migration compared to vehicle treated cells (Fig. 3a). By comparison, MEFs supplemented with 21D1 cell-derived secretome had significantly elevated cell migration (Fig. 3a). Importantly, silencing of MMP1 in the secretome led to a significant reduction in MEF cell migration (Fig. 3a).

To show that MMP1 can enhance MEF cell migration directly, recombinant MMP1 (in addition to the secretome from all three cell lines) was supplemented to MEFs. As expected, addition of recombinant MMP1 elevated MEF cell migration (Fig. 3a, right panel), and in particular, cells supplemented with MDCK secretome plus recombinant MMP1 had significantly higher cell migration. These experiments show that recombinant MMP1 can directly enhance fibroblast migration, and physiological cell-derived secretome samples containing elevated MMP1 levels can also have the same effect.

### Recipient endothelial cell angiogenesis is impaired following addition of 21D1^−MMP1^ cell-derived secretome

Next, we compared the ability of secreted proteins from the three cell lines to regulate angiogenesis of human umbilical vein endothelial cells (HUVECs). Supplementation with MDCK secretome did not change HUVEC tube length formation or number of branch points, compared to vehicle (control) treated cells (Fig. 3b,c). By contrast, addition of 21D1 cell-derived secretome significantly increased both tube length and branching, whilst knock-down of MMP1 attenuated these effects (Fig. 3b,c). Compared to supplementation with cell-derived secretome only, addition of secretome plus recombinant MMP1 slightly elevated HUVEC angiogenesis, although the effects were not statistically significant (Fig. 3b,c).

### Perturbation of MMP1 levels alters composition of the cell-derived secretome

**Figure 4:**
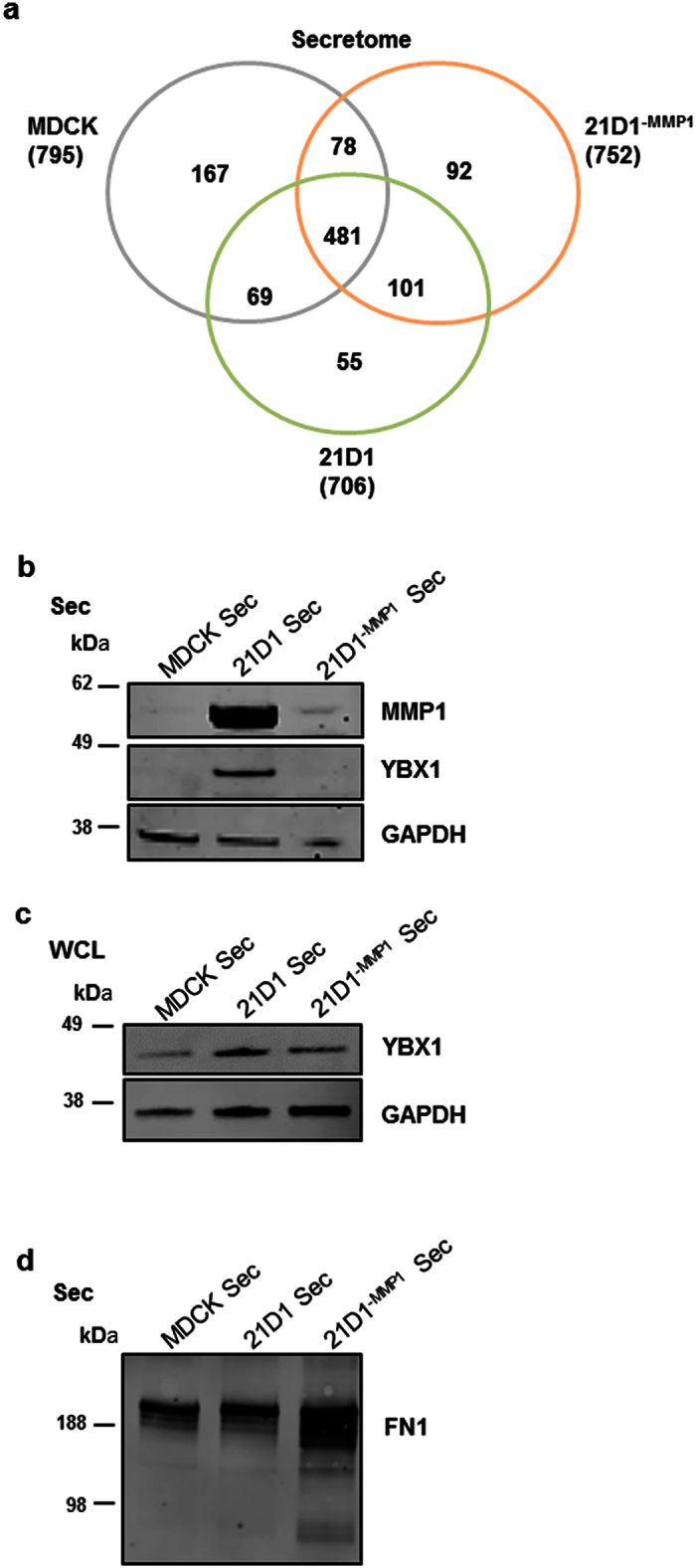
MMP1 remodels the composition of the cell secretome. **(a)** Identification of secretome proteins derived from MDCK, 21D1 and 21D1^−MMP1^ cells. The Venn diagram depicts proteins co-identified in secretome samples. For a complete list, and label-free quantification of proteins that are differentially expressed, see Supplemental Table S1. **(b)** Immuno-blot analysis of MMP1, and YBX1 expression in secretome samples. GAPDH is the loading control. **(c)** MEF cells were supplemented with MDCK, 21D1 or 21D1^−MMP1^ secretome, and expression of YBX1 examined in MEF cell lysates by immuno-blotting. GAPDH is the loading control. **(d)** Immuno-blot analysis of fibronectin (FN1) expression in secretome samples. GAPDH is the loading control.

Our functional assays demonstrated that addition of secretome proteins from cells with elevated MMP1 expression promoted fibroblast migration and endothelial cell angiogenesis. We suspected that this would be due to direct extracellular protein remodelling by MMP1, which would change the composition of the secretome. To profile the protein constituents, secretome from MDCK, 21D1 and 21D1^−MMP1^ cells was subjected to proteomics-based mass spectrometry and label-free quantitative analysis (Supplemental Fig. 3A,C). As expected, peptides and proteins were found to be differentially-expressed between all three secretome samples (Fig. 4a, and Supplemental Table S1 and S2). As secretome comparisons of MDCK versus 21D1 have been analysed previously^30^,^32^, we focussed specifically upon changes that result due to knock-down of MMP1 (21D1 vs 21D1^−MMP1^).

We identified proteins that were significantly down-regulated (Fold change > 2 and p-value < 0.05) in the secretome of 21D1^−MMP1^ cells (higher expression in 21D1 cells), including several which are known to promote EMT and cell migration including CSF-1, Y-box binding protein 1 (YBX1) and DnaJ homolog subfamily A member 1 (DNAJA1) (Supplemental Table S3). This may explain the reduced oncogenicity observed in 21D1^−MMP1^ cells. We have previously reported the elevated expression of YBX1 in the 21D1 secretome^33^, and that increased expression can promote angiogenesis^34^. We subjected the secretome samples to western blotting to confirm that diminished MMP1 expression correlated with reduced YBX1 levels (Fig. 4b). Furthermore, we verified that elevated YBX1 levels in the 21D1 cell secretome could be transferred to recipient MEF cells. Following secretome supplementation, western blot analysis of MEF cellular lysates validates increased YBX1 expression (Fig. 4c). Additionally, proteins involved in angiogenesis such as IL1R1, MAP1B and ANP32B were identified to be significantly elevated in the 21D1 secretome (Supplemental Table S3).

We also identified proteins significantly upregulated in the 21D1^−MMP1^ secretome, compared to the 21D1 secretome (Supplemental Table S4). Most significantly, was the strong expression of Wnt inhibitor NOTUM which is known to represses cell migration^35^,^36^, as well as constituents mediating cell-cell adhesion (EpCAM, CDH3 and CDH16) and cell-matrix contact (FRAS1 and TNS1). The regulation of these proteins may shed light on why 21D1^−MMP1^ cells exhibit reduced cell migration and invasiveness.

### MMP1 silencing reduces proteolytic processing of secretome substrates

We were interested in discovering novel MMP1 substrates, and predicted that reduced MMP1 expression and proteolytic activity may cause accumulation of its protein substrates in the 21D1^−MMP1^ secretome. We mined our data for known MMP1 substrates^37^, and found that COL1A2, CTGF, HSPG2 and fibronectin (FN1) had elevated expression in 21D1^−MMP1^ secretome, compared to 21D1 secretome (Supplemental Table S2). Elevated expression of FN1 in the 21D1^−MMP1^ secretome was validated by western blotting (Fig. 4d).

**Figure 5:**
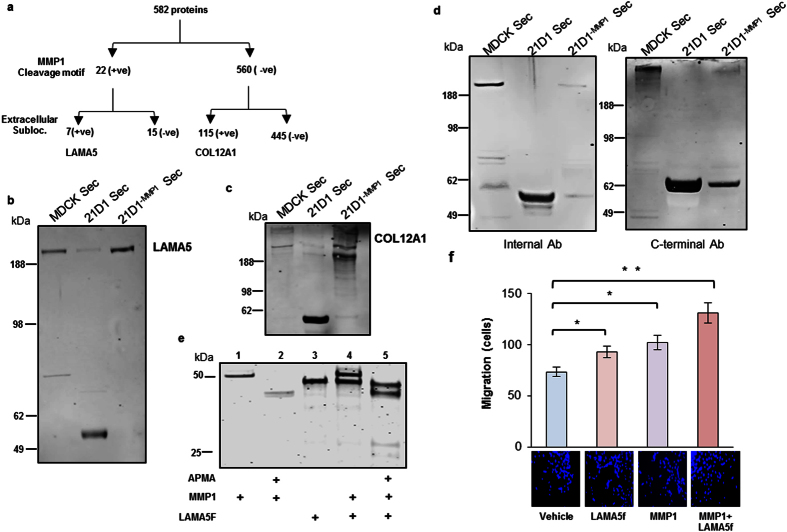
LAMA5 is proteolytically fragmented by MMP1. **(a)** *In silico* prediction of MMP1 substrates. Candidates were first screened for potential MMP1 cleavage recognition motif, and then analzyed for extracellular localization. **(b**,**c)** Immuno-blot analysis of LAMA5 **(b)**, and COL12A1 **(c)** expression in secretome samples. **(d)** Immuno-blot analysis of LAMA5 fragments in cell-derived secretome samples. Left Panel: Blot probed with antibody recognising an internal region of the protein. Right Panel: Blot probed with antibody recognising the C-terminus of the protein. **(e)** *In vitro* cleavage of a recombinant LAMA5 fragment (LAMA5f) derived from the C-terminus of the protein, by recombinant MMP1. MMP1 was activated by APMA and reacted with LAMA5f, generating proteolytic products. SDS was added to terminate all reactions, and products subjected to electrophoresis, followed by Sypro-ruby gel staining and immunofluorescence imaging. **(f)** MEF cells (5 × 10^4^) were supplemented with recombinant C-terminal LAMA5f (1 μg), APMA activated recombinant MMP1 (100 ng), or LAMA5f pre-incubated with MMP1 to generate proteolytic products. MEF cell migration was examined by transwell assay over 24 hr. Transversing cells were stained with DAPI and imaged and counted (*n* = 3; average ± sem; *P < 0.05, **P < 0.01).

We also reasoned that if MMP1 protease activity was attenuated, then perhaps the overall abundance of a substrate in the secretome would not change, but only the degree of processing. To explore this further, we subjected the 582 proteins identified in both 21D1 and 21D1^−MMP1^ secretome samples (Fig. 4a) to ScanProsite prediction (http://prosite.expasy.org/scanprosite/), based on the known cleavage MMP1 motif^38^. This *in silico* analysis predicted 22 candidate proteins to be cleaved by MMP1 (Fig. 5a, and Supplemental Table S5). Focussing on the 7 proteins with extracellular localization, LAMA5 was selected for western blot validation. While we observed the full length protein (400 kDa) in the MDCK secretome, we detected a ~55 kDa fragment in the 21D1 secretome (Fig. 5b). Furthermore, LAMA5 processing was attenuated in the 21D1^−MMP1^ secretome (Fig. 5b). We also explored the possibility, the *in silico* prediction could have false negatives, and therefore biological substrates could also be in the list of 560 proteins not predicted by the software (Fig. 5a). Following a similar logic to LAMA5, collagen alpha-1(XII) chain (COL12A1) was selected for further proteolytic processing confirmation by western blot. Once again, a fragment ~60 kDa in size was only observed in the secretome of 21D1 cells (Fig. 5c), indicating possible processing by MMP1. Together, LAMA5 and COL12A1 potentially represent novel MMP1 substrates.

### LAMA5 is proteolytically processed by MMP1

Full length LAMA5 is comprised of 3695 amino acids (~400 kDa) and contains several functional domains (Supplemental Fig. 4), including the coiled-coiled region and various laminin G-like domains (LG1–LG5). Towards characterising the ~55 kDa LAMA5 fragment identified in the 21D1 secretome, we probed the secretome samples again with specific antibodies corresponding to an internal (epitope between residues 1380–1540) and C-terminal region of the protein (epitope between residues 3600–3695). Strikingly, two independent LAMA5 fragments (an internal ~55 kDa, and C-terminal ~62 kDa) were detected in 21D1 secretome using both reagents, and the degree of processing reduced in 21D1^−MMP1^ secretome (Fig. 5d). This indicates that MMP1 can specifically generate at least two distinct LAMA5 proteolytic fragments.

We next investigated whether MMP1 could cleave LAMA5 directly *in vitro* using recombinant proteins. Proteins and products were run on SDS-PAGE and stained with SYPRO Ruby for visualization with fluorescence (Fig. 5e). Full length recombinant LAMA5 was not available given its size, however we purchased a C-terminal fragment corresponding to residues 3400–3695 termed LAMA5f. MMP1 (Fig. 5e, Lane 1) was activated by APMA^39^,^40^ and as expected, we observed a reduction in molecular weight corresponding to the loss of its propeptide domain (Lane 2). LAMA5f appeared as a single band at ~52 kDa (Lane 3), and addition of activated MMP1 resulted in proteolytic products observed around ~25–28 kDa (Lane 5). Note, that when LAMA5f was reacted with non-activated MMP1, the proteolytic products were not generated (Lane 4), demonstrating specific MMP1 activity.

To test the biological activity of these MMP1-derived LAMA5f products, we examined MEF cell migration using transwell assay. Addition of activated MMP1 or LAMA5f increased MEF cell migration (Fig. 5f). However, when MMP1 and LAMA5f were preincubated to generate the ~25–28 kDa products subsequently added to MEF cells, MEF cell migration was heightened most significantly (Fig. 5f). This indicates that MMP1 can process LAMA5, and these fragments promote recipient MEF cell motility.

### LAMA5 fragments promote angiogenesis of endothelial cells via integrin receptor and ERK signalling

**Figure 6:**
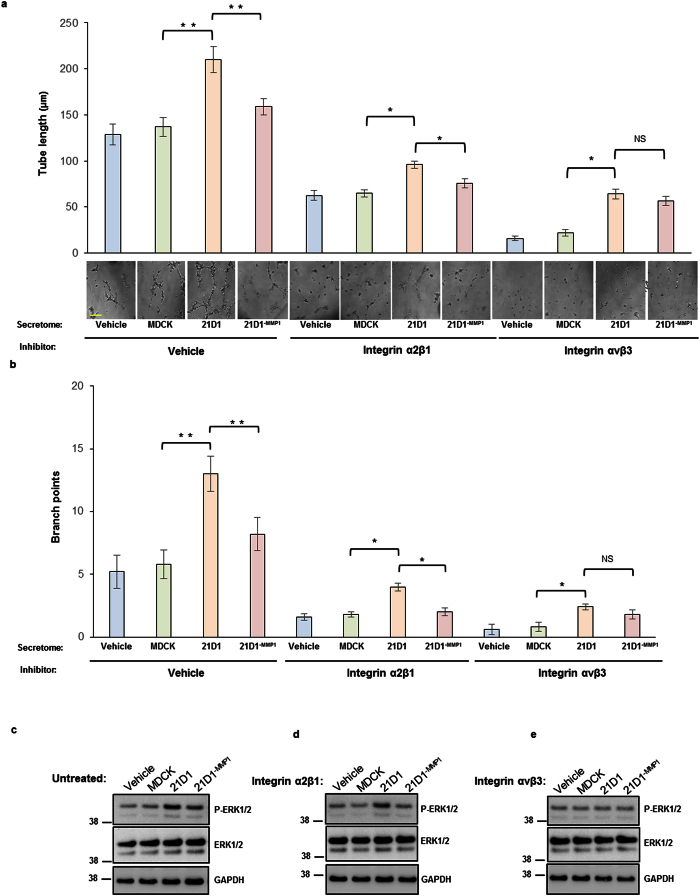
21D1 cell-derived secretome promotes HUVEC angiogenesis through integrin αvβ3. HUVECs (7 × 10^4^) were pre-treated with various integrin inhibitory antibodies (20 μg/ml) for 1 hr, followed by supplementation with corresponding secretome samples. After 24 hr, tube formation **(a)** and branch points **(b)** were examined, imaged, and quantified. Scale bar = 50 μm. (representative images from *n* = 3, *P < 0.05, **P < 0.01 and NS = no significant difference). **(c**–**e)** Immuno-blot-based determination of ERK1/2 signalling in HUVECs following pre-treatment with or without integrin inhibitors, and supplementation with various cell-derived secretome samples. Phosphorylated ERK1/2 (p-ERK1/2) represents active signal, and GAPDH used as loading control.

Finally, we investigated possible molecular mechanisms by which MMP1-processed LAMA5 in the 21D1 secretome could promote angiogenesis in recipient HUVECs. Based on previous reports that LAMA5 interacts with various integrin receptors^41^,^42^, we tested the ability of inhibitory integrin antibodies to block receptors activated by the 21D1 secretome. Compared to cells supplemented with MDCK secretome, supplementation with 21D1 secretome increased HUVEC tube length and branching, whilst 21D1^−MMP1^ secretome supplementation attenuated these effects sightly (Fig. 6a,b, left panels). Pre-treatment of HUVECs with anti-integrin α2β1 inhibitors prior to secretome addition decreased overall tube length and branching, however addition of 21D1 secretome still significantly elevated HUVEC angiogenesis compared to 21D1^−MMP1^ secretome (Fig. 6a,b, middle panels). By comparison, pre-treatment of HUVECs with anti-integrin αvβ3 inhibitors restored the levels of angiogenesis observed following 21D1 secretome addition to levels observed for cells supplemented with 21D1^−MMP1^ secretome (Fig. 6a,b, right panels).

Finally, we tested the pathways in HUVECs that may be active following secretome supplementation. In untreated cells, supplementation with 21D1 secretome elevated p-ERK1/2 levels compared to HUVECs supplemented with MDCK or 21D1^−MMP1^ secretome (Fig. 6c). Similarly, p-ERK1/2 levels were highest in HUVECs treated with anti-integrin α2β1 inhibitors (Fig. 6d). However, treatment with anti-integrin αvβ3 inhibitors restored p-ERK1/2 levels of HUVECs supplemented with 21D1 secretome to that of cells supplemented with MDCK or 21D1^−MMP1^ secretome (Fig. 6e). Together, these experiments demonstrate that increased MMP1 and LAMA5 processing in the 21D1 secretome can promote angiogenesis of recipient HUVECs via integrin αvβ3 and downstream ERK1/2 signalling.

## Discussion

Secretion of MMPs from cancer cells have been described as a mechanism that facilitates cancer progression via modification of the tumour microenvironment^43^. Consistent with the findings that MMPs can promote EMT^44^, we have previously reported that MDCK cells transformed with H-Ras (21D1 cells) significantly induce MMP1 expression^30^,^32^. To explore the functional significance, in the current study we knocked-down expression of MMP1 in 21D1 cells and examined changes to their oncogenic phenotype. Diminished expression of MMP1 in 21D1 cells reduced cell migration, invasion, and anchorage-independent growth *in vitro*. When these cells were grown as a subcutaneous xenograft in mice, the tumour volume was significantly compromised. This could be due to effects on the oncogenic cells themselves, or by influencing cells in the tumour microenvironment (fibroblasts and endothelial cells). Depletion of MMP1 from the 21D1 cell secretome impeded recipient fibroblast cell migration, and also impaired endothelial cell angiogenesis. Both of these effects may have contributed to reduce growth of the tumour xenograft *in vivo*.

To shed light on potential molecular mechanisms, we profiled the protein composition of the secretome using proteomics and also employed *in silico* prediction of candidate MMP1 substrates, followed by western-blot validation. Our analysis revealed that MMP1 can modulate the composition of the extracellular microenvironment by: 1) increasing expression of pro-oncogenic proteins and proteins promoting cell migration, 2) decreasing expression of proteins mediating cell adhesion and, 3) regulating surrounding cells by proteolytic processing of ECM proteins. We discovered that LAMA5 is a biological substrate of MMP1, and its proteolytic activity can produce at least two different fragments in the secretome from cells undergoing Ras-induced EMT. Given laminins are major contributors to basement membrane architecture^45^, the functional importance of LAMA5 proteolytic processing by MMP1 includes reduced structural rigidity of the ECM. Moreover, as the LAMA5 subunit is a component of at least four laminin heterotrimers (laminin-10, -11, -15 and α5β2γ2)^46^, its processing by MMP1 could potentiate reduced stability of the basement membrane. It is likely that this contributes to the enhanced motility and invasion of oncogenic cells such as 21D1 cells and MEFs.

**Figure 7:**
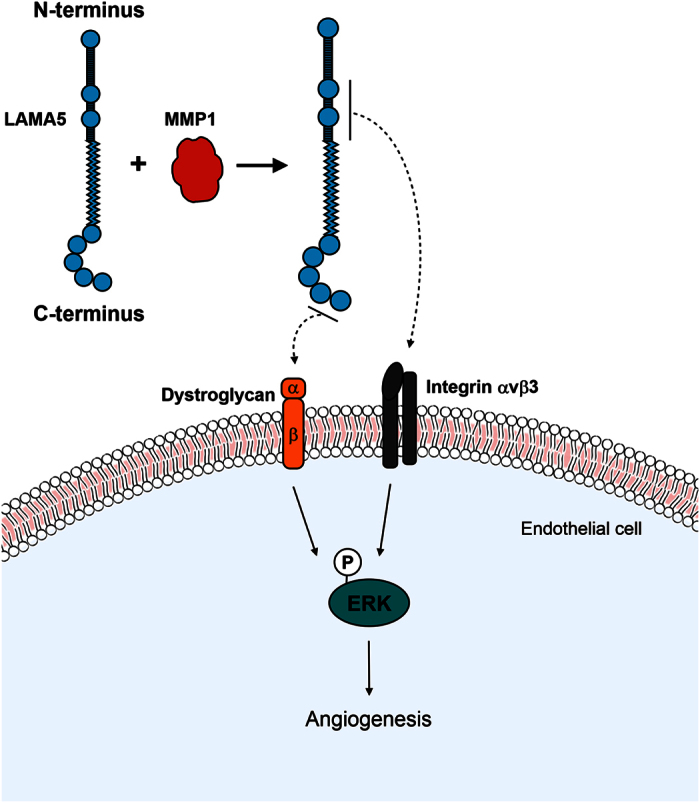
Proteolytic processing of LAMA5 by MMP1 promotes endothelial cell angiogenesis. Cartoon depiction of LAMA5 internal- and C-terminal fragments generated by MMP1. Protease activity releases fragments with biological activity that can act on recipient cells residing in the tumour microenvironment. Binding of the internal fragment to integrin αvβ3 on HUVECs promotes angiogenesis via downstream ERK1/2 signalling. Binding of the C-terminal fragment to dystroglycan on endothelial cells may be a concurrent mechanism to initiate angiogenesis.

LAMA5 has also been reported to be cleaved by membrane-type 1 MMP (MMP14), a transmembrane protease that can promote the cell migration of prostate cancer cells via cleavage of LAMA5 in laminin-10^47^. As MMP14 is tethered to the cell surface and therefore is restricted to the proximal microenvironment, increased secretion of MMP1 by oncogenic cells may provide a mechanism to regulate cells and ECM proteins at sites distal to the developing tumour. Moreover, our findings also implicate the release of MMP1-derived LAMA5 fragments with bioactive activity, into the tumour microenvironment. We demonstrate for the first time that knock-down of MMP1 remodels the secretome to reduce recipient endothelial cell branching and sprouting. It is still unclear how many LAMA5 fragments are produced by MMP1, and the precise cleavage sites. However, we detected an internal ~55 kDa fragment, and a C-terminal ~62 kDa fragment. We anticipate that these fragments interact with multiple targets on the endothelial cell membranes (Fig. 7). For example, the C-terminal derived fragments containing the LG domains interact with α-dystroglycan, the N-terminus with integrin α3β1, and an internal region interacts with αvβ3^48^. Indeed, the internal fragment we detected contains two RGD sequence motifs, and therefore likely to mediate binding to αvβ3^49^, an integrin known to promote angiogenesis^50^. Furthermore, 21D1 secretome activated ERK1/2 signalling in recipient HUVECs, and this is a well known axis that induces an angiogenic response^51^,^52^.

The biological significance of our findings is reflected by the fact αvβ3 is involved in various facets of tumour progression. Integrin αvβ3 is expressed on a variety of cancer cells, and promotes tumour cell adhesion, proliferation, migration and invasion, and has been the molecular target of several clinical trials^53^. Therapeutic antibodies Vitaxin and Abegrin, and inhibitory peptide Cilengitide have been tested as clinical anti-tumour agents, however none have progressed to FDA approval due to various problems to demonstrate efficacy, decrease significant tumour response, or significantly improve patient survival^53^,^54^. Given a developing tumour requires blood vessels to deliver oxygen and nutrients, blocking tumour angiogenesis remains a hopeful strategy. Moreover, integrins are still touted as essential clinical targets for which we must develop effective agents and extend our knowledge of tumour–ECM interactions^54^. Therefore in the future, simultaneous inhibition of integrin αvβ3 with MMP1 and LAMA5 may provide greater efficacy, and an alternate approach to destabilize tumour vasculature.

Epithelial cells undergoing Ras-induced EMT secrete elevated levels of MMP1. This leads to extensive remodelling of the secretome, and alters expression of ECM constituents that modulate cell adhesion, migration and invasion. In addition, MMP1 regulates cleavage of the basement membrane protein LAMA5, and proteolytic processing results in the generation of LAMA5 fragments which stimulate fibroblast cell migration, and promote endothelial cell angiogenesis. The latter is mediated by integrin αvβ3 on the endothelial cell surface and the ERK1/2 signalling pathway is involved in transducing the angiogenic signal.

## Methods

### Cell culture

MDCK^32^, 21D1^30^,^32^, 21D1^−MMP1^, MEF (ATCC) and HUVECS (ATCC) were maintained at 37 °C with 10% CO_2_ in high-glucose Dulbecco’s Modified Eagle’s Medium (DMEM) (Life Technologies, Carlsbad, CA, USA) containing 10% (v/v) Fetal Calf Serum (Life Technologies), 1% (v/v) Penicillin Streptomycin (Pen/Strep) (Life Technologies).

### Generation of 21D1^−MMP1^ cells

Customized Pre-miR (Life Technologies) targeting the *Canis familiaris* MMP1 transcript (Ensembl accession number ENSG00000196611), was designed using the Invitrogen RNAi Designer (GeneArt Synthesis). Stable cell lines were generated following manufacturer’s instructions.

### Phase contrast microscopy

Cells (1 × 10^6^) were washed with DMEM and imaged on an inverted Nikon Eclipse TE300 microscope equipped with a 10x objective (Nikon Plan Fluor) in phase-contrast mode using an attached 12.6mp digital camera (Nikon DXM1200C). Images obtained from MDCK, 21D1, and 21D1^−MMP1^ cells (5 independent fields of view) were processed with Nikon Elements Imaging Software (v.3.00, SP$ (Build 502)).

### Protein quantification and immunoblotting

Protein quantification was performed using 1D-SDS-PAGE/SYPRO Ruby protein staining densitometry, as previously described^33^. For immunoblotting (10 μg), membranes were probed with primary antibodies [mouse anti-CDH1 (BD Biosciences; 1:1000), rabbit anti-MMP1 (Santa Cruz Biotechnology; 1:1000), mouse anti-VIM (Cell signalling; 1:1000), rabbit anti-YBX1 (Abcam; 1:1000), mouse anti –FN1 (Sigma, 1:1000), rabbit anti-LAMA5 (Santa Cruz Biotechnology; 1:1000), rabbit anti-COL12A1 (Santa Cruz Biotechnology; 1:1000), rabbit anti-p44/42 MAPK (ERK 1/2) (Cell signalling; 1:1000), rabbit anti-phospho-p44/42 MAPK (p-ERK 1/2) (Cell Signalling; 1:1000), or mouse anti-GAPDH (Life technologies; 1:12,000)] for 1 hr at RT in TTBS (50 mM Tris, 150 mM NaCl, 0.05% (v/v) Tween 20 in PBS) followed by incubation with the corresponding secondary antibodies (IRDye 800 goat anti-mouse IgG or IRDye 700 goat anti-rabbit IgG (1:15,000, LI-COR Biosciences) for 1 hr at RT in TTBS. Immunoblots were imaged and visualised using the Odyssey Infrared Imaging System, (v3.0, LI-COR Biosciences, Nebraska USA).

### Cell line characterization assays

The proliferation assay was performed as previously described^55^. Cell growth assays were performed as previously described^34^. The wound healing/scratch^56^, migration^34^, invasion^34^, and soft agar growth assays^34^, were performed as previously described. All assays were performed in triplicate.

### Tumour xenografts and immunofluorescence

Animal care and experiments conducted adhered to the guidelines of the Australian Code of Practice for the Care and Use of Animals for Scientific purposes, endorsed by the National Health and Medical Research Council. All experimental protocols were approved by our institutional animal ethics committee at La Trobe University (AEC12-50). For xenograft assays, experiments were performed as previously described^34^,^57^. Briefly, cells (1 × 10^6^ cells/site) were injected subcutaneously into NOD/SCID male mice (n = 8) in both inguinal regions, and primary tumour volumes calculated at specified times according to V = (small diameter)^2^ × (larger diameter) × 0.5^58^. Animals were sacrificed 5 weeks post-injection, tumours excised and fixed in 4% paraformaldehyde, and paraffin embedded for histological examination. Fresh sections (20 μm) were prepared using a vibratome (Leica VT 1000S) and immuno-stained as previously described^59^. Incubation with primary antibody (anti-CD31 (Abcam 1:50)) was performed overnight at 4 °C, and secondary incubation using AlexaFluor 488-conjugated goat anti-mouse IgG (Life Technologies). Nuclei were stained with To-pro-3 (1:1000), and imaging performed using a Zeiss LSM 780 confocal microscope (63x magnification).

### Secretome preparation

Secretome samples were prepared as previously described^34^,^57^.

### Proteomic analysis

Proteomic analyses were performed using 20 μg secretome sample in biological duplicates, as previously described^34^, with slight amendments. Secretome samples obtained from MDCK, 21D1 and 21D1^−MMP1^ cells were lysed in SDS sample buffer (4% (w/v) SDS, 20% (v/v) glycerol, 0.01% (v/v) bromophenol blue, 0.125 M Tris-HCl, pH 6.8), proteins separated by SDS-PAGE, and visualized by Imperial Protein Stain (Thermo Fisher Scientific). The entire gel lane was excised as multiple gel bands, and destained (50 mM ammonium bicarbonate/acetonitrile), reduced (10 mM DTT (Calbiochem) for 30 min), alkylated (50 mM iodoacetic acid (Fluka) for 30 min) and trypsinized (0.4 μg trypsin (Promega Sequencing Grade) for 16 h at 37 °C), as described^60^.

Peptides were desalted using reverse-phase C18 StageTips^61^, and eluted in 85% (v/v) acetonitrile (ACN) in 0.5% (v/v) formic acid (FA). Peptides were lyophilised in a SpeedVac and acidified with buffer containing 0.1% FA, 2% ACN. A nanoflow UPLC instrument (Ultimate 3000 RSLCnano, Thermo Fisher Scientific) was coupled on-line to an Orbitrap Elite mass spectrometer (Thermo Fisher Scientific) with a nanoelectrospray ion source (Thermo Fisher Scientific). ~3 μg peptide was loaded per injection (Acclaim PepMap100 C18 5 μm 100 Å, Thermo Fisher Scientific), and separated (Vydac MS C18-RP column, 25 cm, 75 μm inner diameter, 3 μm 300 Å, Grace, Hesperia, CA) with a 120-min linear gradient from 0–100% (v/v) phase B (0.1% (v/v) FA in 80% (v/v) ACN) at a flow rate of 250 nL/min. Operation of the Orbitrap Elite have been described previously^34^.

### Database searching and protein identification

Raw data was processed using MaxQuant^62^ (v1.5.3.12) searched with Andromeda (v1.5.3.8) against a Uniprot canine database with 28698 entries (Aug-2015). Data was searched as described^34^, with tryptic digestion and allowance of up to 2 missed cleavages, with a parent mass tolerance of 10 ppm, fragment tolerance of 0.5 Da, minimum peptide length 7 residues, and carbamidomethyl (C) specified as a fixed modification, and oxidation (M) and N-terminal acetylation as variable modifications. A 1% FDR was used at the peptide and protein levels, with reported proteins containing at least 2 unique peptide identifications (Supplemental Tables S1 and S2). Data was analysed with label-free quantitation (LFQ) with min ratio count of 2^63^. LFQ intensity values were normalized for protein length and fold change ratios calculated. Contaminants and reverse database identifications were excluded from data analysis. Proteins commonly identified in both biological replicates were used to compare protein expression between secretome samples. Raw mass spectrometry data is deposited in the PeptideAtlas and can be accessed at http://www.peptideatlas.org/PASS/PASS00794.

### MMP1 proteolytic cleavage assays

The ability of MMP1 to cleave LAMA5 was validated using recombinant proteins. 0.05 μg/ul MMP1 (R&D Systems) was activated with (1 mM) 4-aminophenylmercuric acetate (APMA) (Sigma) at 37 °C for 2 hr, according to the manufacturer’s instructions^39^. Next, activated MMP1 (0.25 μM) was added to 0.75 μM LAMA5 (CUSABIO, C-terminal fragment corresponding to amino acids 3400–3692), and incubated at 37 °C for 6 hr. Reactions were terminated by addition of 4xSDS sample buffer, and all products analysed by SDS-PAGE, followed by SYPRO Ruby (Invitrogen) staining, according to the manufacturer’s instructions. Proteins were visualized using a Typhoon 9410 variable mode imager (Molecular Dynamics) using a 532 nm excitation laser and a 610BP30 emission filter at 100 μm resolution.

### Endothelial cell tube formation assay

Endothelial cell tube formation assays were performed as previously described^64^. Briefly, HUVECs (7 × 10^4^ cells/well) were resuspended in DMEM + 5% Fetal Bovine Serum (FBS), and seeded onto growth factor-reduced BD matrigel (1 mg/ml) (96-well) for 1 hr, then supplemented with 30 μg secretome sample for 24 hr. Tube-like structures were imaged using Nikon Eclipse TE300 microscope, branch points were manually counted from 5 random fields of view and tube length measured and quantified. For integrin neutralisation assays, HUVECs (7000 cells/well) were pre-treated with 20 μg/ml αvβ3 or α2β1inhibitory antibody (Millipore) for 1 hr, prior to supplementation with 30 μg secretome.

### Experimental Design and Statistical Rationale

Functional cell biological assays were conducted by a minimum of 3 independent biological experiments. For all assays, statistical analysis was performed using Student’s t-tests using GraphPad Prism (version 5.0), with *p < 0.05 and **p < 0.01 considered statistically significant. *In vivo* tumour xenograft experiments were performed using 8 animals per condition. Mass spectrometry analysis of secretome proteins was performed in biological duplicates, and only proteins identified in both replicates used for label-free quantification. Statistical testing of proteomic data was performed using a Poisson distribution with EdgeR software (version 3.2), with *p < 0.05 considered statistically significant^65^. Furthermore, selected proteomic findings were validated using orthogonal approaches including western immuno-blotting, recombinant protein studies, and inhibitory antibody blocking assays.

## Additional Information

**How to cite this article**: Gopal, S. K. *et al.* Transformed MDCK cells secrete elevated MMP1 that generates LAMA5 fragments promoting endothelial cell angiogenesis. *Sci. Rep.* **6**, 28321; doi: 10.1038/srep28321 (2016).

## Supplementary Material

Supplementary Information

Supplementary Dataset
