# Supplementary material for: Transformed MDCK cells secrete elevated MMP1 that generates LAMA5 fragments promoting endothelial cell angiogenesis
Source: Sci Rep. 2016 Jun 21;6:28321. doi: 10.1038/srep28321 (PMC4914959; doi:10.1038/srep28321)
Supplement: Supplementary Information [file srep28321-s1.pdf]

Transformed MDCK cells secrete elevated MMP1 that generates LAMA5 fragments promoting endothelial cell angiogenesis

Shashi K. Gopal[1], David W. Greening[1], Hong-Jian Zhu[2], Richard J. Simpson[1], and Rommel A. Mathias[3,4*]

[1] Department of Biochemistry and Genetics, La Trobe Institute for Molecular Science, La Trobe University, Melbourne, Victoria 3086, Australia

[2] Department of Surgery, The University of Melbourne, Royal Melbourne Hospital, Melbourne, Victoria 3050, Australia

[3] Department of Biochemistry and Molecular Biology, Biomedicine Discovery Institute, Monash University, Clayton, Victoria 3800, Australia

[4] Department of Microbiology, Biomedicine Discovery Institute, Monash University, Clayton, Victoria 3800, Australia

*** To whom correspondence should be addressed:**

Dr. Rommel A. Mathias

Biomedicine Discovery Institute

Monash University

23 Innovation Walk

Clayton VIC 3800

Australia

T: +61 3 9902 9322

E: rommel.mathias@monash.edu

S1A

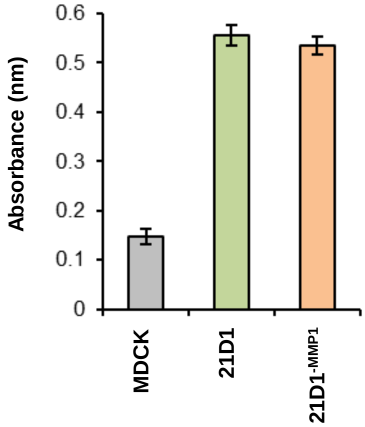

S1B

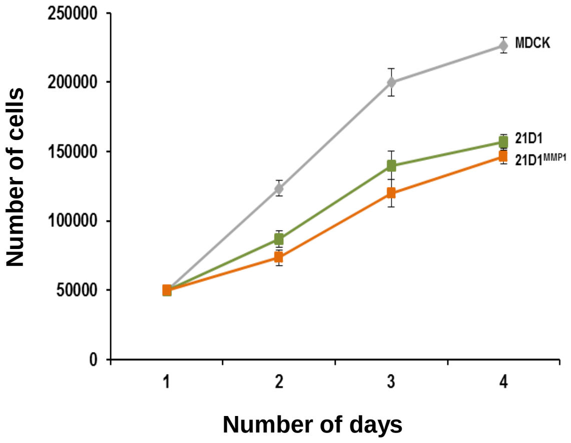

(S1A) Metabolic activity of cell lines by MTT assay over 24h (n=3; average±sem). **(S1B)** Determination of cell growth by counting sub-confluent cell numbers every 24 hr over 4 days. (n = 3; average ± SEM).

S2

MDCK,21D1 and 21D1^{-MMP1} cells

↓

Serum-free culture (24 h)

↓

Differential Centrifugation
480 *g*, 5 min
2000 *g*, 10 min

↓

Centrifugal Ultrafiltration concentrated to 1 mL
(3K NMWL)
concentrate culture medium

↓

(MDCK,21D1 and **21D1^{-MMP1}** ***Secretome)***

(S2) Workflow outlining isolation of secretome from MDCK, 21D1 and 21D1^{-MMP1} cells.

S3A

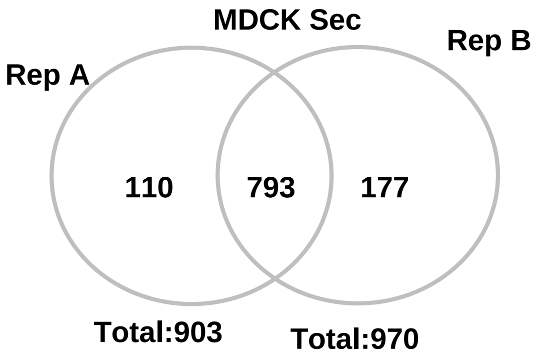

S3B

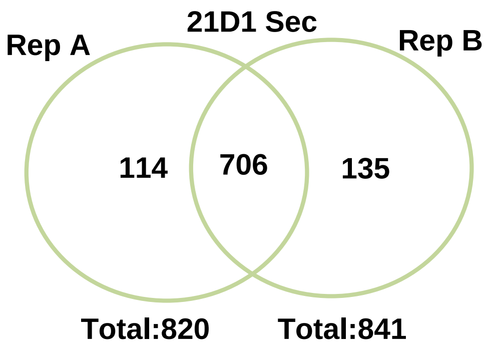

S3C

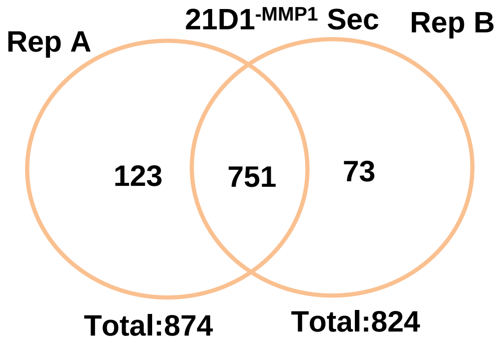

(S3) Venn diagrams showing reproducibility of proteins identified in the secretome samples by mass spectrometry. Analysis of two replicates were performed. See Supplemental Table S1.

S4

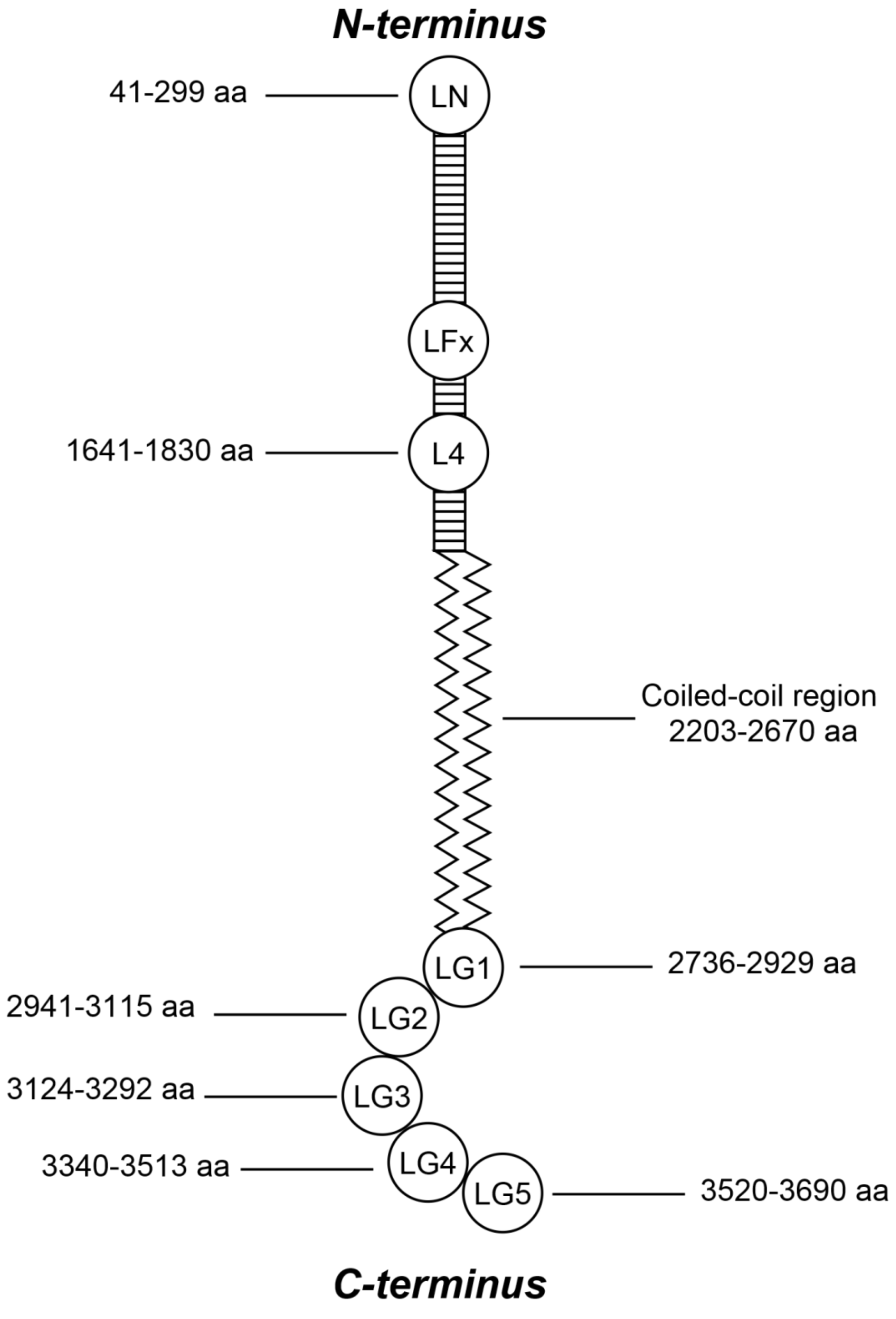

(S4) Schematic representing known LAMA5 domains. Location of LN domain at the N-terminal region, and LG domains (LG1-LG5) at the C –terminal region of LAMA5. In silico prediction of LAMA5 cleavage based on MMP1 recognition motif.
